# Synthesis of Mesoporous Silica Imprinted Salbutamol with Two TEOS/MTES Ratio Compositions through the Direct Incorporation Method for Salbutamol Separation

**DOI:** 10.1155/2023/2871761

**Published:** 2023-01-30

**Authors:** Ike Susanti, Mutakin Mutakin, Aliya Nur Hasanah

**Affiliations:** ^1^Department of Pharmaceutical Analysis and Medicinal Chemistry, Faculty of Pharmacy, Universitas Padjadjaran, Sumedang, Indonesia; ^2^Drug Development Study Center, Faculty of Pharmacy, Universitas Padjadjaran, Sumedang, Indonesia

## Abstract

Molecularly imprinted mesoporous silica (MIPMS) is one of the methods to improve site accessibility molecule target on molecularly imprinted polymer (MIP) for application in solid-phase extraction (SPE). The MIPMS was prepared using salbutamol sulfate as template molecule, cetyltrimethylammonium bromide as a directing agent, and tetraethyl orthosilicate and methyltriethoxysilane were used as silica precursor and organosilane. In this study, two TEOS : MTES ratios were used. The MIPMS-2 with 3 : 1 ratio of TEOS : MTES has better analytical performance than the MIPMS-1 with 2 : 1 ratio of TEOS : MTES. The adsorption capacity of MIPMS-2 was about 0.0934 mg/g, and it was 0.0407 mg/g for NIPMS-2. The extraction ability of MIPMS-2 was good, with a recovery of about 104.79% ± 1.01% of salbutamol in spiked serum. The imprinting factor (IF) value obtained is 1.2. When serum was spiked with salbutamol and terbutaline, the ability of NIPMS-2 to recognize salbutamol increased. Therefore, optimizing the conditions for the MIPMS synthesis is necessary to produce a sorbent with better selectivity.

## 1. Introduction

Salbutamol is a short-acting beta-2 agonist (SABA) drug used to treat and prevent bronchospasm in patients with reversible obstructive airways disease [1]. Analysis of salbutamol in the biological samples is necessary to monitor therapeutic doses in severe acute asthma [2]. It is difficult for pediatric or adult patients to distinguish between a failed response to treatment due to an insufficient dose or increased salbutamol toxicity [3].

Sample preparation is one of the essential steps in complex matrix analysis. Appropriate sample preparation methods will not only ensure that the analyte is suitable for the detection limits of the instrument but also increase the method's selectivity by eliminating unnecessary and interfering compounds from the matrix [4]. Solid phase extraction (SPE) is the most widely used method for separating the analyte in liquid samples [5, 6]. However, SPE can be used as a preparation method for solid samples, but it requires to be pretreated before processing in SPE [7, 8]. The advantages of the SPE method are relatively simple, have high efficiency, and requires small amount of the sample. Suryana et al. compared the analytical performance between conventional C-18 cartridge and polymerly imprinted polymer (MIP) as an SPE sorbent for extracting salmeterol xinafoate (SLX) from human serum. The recoveries obtained using the MIP sorbent and the conventional C-18 cartridge were 92.17% ± 2.66% and 9.11% ± 2.96%, respectively. Based on these results, conventional sorbents are less selective than MIP sorbents. [9]. Molecularly Imprinted Solid Phase Extraction (MI-SPE) is a solid extraction method with good selectivity and can provide an active site or catalytic site that can bind to molecular targets. Molecularly imprinting polymers (MIP) are synthetic polymers with artificial recognition that are complementary in size, shape, and spatial arrangement of the functional group to a template [10]. Synthesis of MIPs is based on formation of a complex between a molecule target and functional monomer. In the presence of an excess crosslinker, a three-dimensional polymer is formed [11]. The template is then removed by cleavage of the corresponding covalent bond from the polymer, and it leaves the enhanced selectivity cavities for rebinding with the molecule template [12]. However, the MIP-SPE method still has drawbacks, such as poor accessibility to molecular targets [13], so surface molecularly imprinting (SMIP) was developed [14].

In the SMIP method, the molecular recognition site is placed on a surface of the support material that can improve binding kinetics, binding capacity, and accessibility of the binding site [15]. Mesoporous silica was used as a support material in the synthesis of SMIP because it has good mechanical and physical properties and modified surface properties. After all, it has abundant active Si-OH bonds [16, 17]. Molecularly imprinted mesoporous silica (MIPMS) has been developed to increase the efficiency, the adsorption capacity, and the accessibility of target molecules to reach the binding site [18, 19].

Recently, a direct incorporation method for MIPMS synthesis was developed. This method is an imprinted method carried out by directly adding the template, surfactant, and silica precursor. This method has several advantages, including easy procedure, uniform distribution of functional groups in pore channels, and increased capacity and selectivity [20]. Silica precursor is one of the materials used to synthesize mesoporous silica. The commonly used silica precursor is tetraethyl orthosilicate (TEOS) [18, 19]. The amount of TEOS affects the mesoporous structure formed. The mesoporous structure generally becomes irregular with a higher concentration of TEOS, while a lower amount will cause the mesoporous structure to not be formed [21]. Methyltriethoxysilane (MTES) is a substance added to increase the hydrophobicity of the surface in the presence of the -CH3 group and can prevent leaching [22]. Comparison of the molar concentrations between MTES and TEOS in the synthesis of MIPMS can affect the adsorption capacity of a MIPMS [20]. Until now, the methods used for the synthesis of MIPs for salbutamol were precipitation polymerization [23, 24] and bulk polymerization [25–27]. Development of MIP for salbutamol using MIPMS with the direct corporation is still not developed, whereas this method has many advantages. These advantages include mesoporous silica in MIPMS which has a rigid structure that is very suitable for forming fine recognition cavities [25, 26, 28]. In addition, MIPMS can provide better accessibility and high selectivity for the target molecule. The binding kinetic for the target molecule in MIPMS is fast because of the thin wall thickness and its mesoporous structure [27]. Therefore, this study synthesized the MIPMS sorbent for rapid preconcentration of salbutamol in serum samples using two TEOS : MTES ratios by the direct incorporation method.

## 2. Materials and Methods

### 2.1. Materials

Salbutamol sulphate was purchased from Supriya Lifescience Ltd., India. Cetyltrimethylammonium bromide (CTAB), tetraethyl orthosilicate (TEOS), and methyltriethoxysilane (MTES) were purchased from TCI. Terbutaline sulfate was obtained from LKT Lab. Sodium hydroxide (NaOH), ethanol, and HPLC grade methanol were obtained from Merck. HPLC grade acetonitrile was purchased from J. T Backer. If not otherwise specified, all chemicals are of analytical grade. The UV-visible spectrophotometer (Analytical Jena Specord 200 using a 1.0 cm quartz cell) is used for measurement of UV absorbance. Identification of the functional group was analyzed by Fourier Transform Infrared (FTIR) IR (Prestige-21 Shimadzu). Analyses of salbutamol in serum after extraction were performed using UPLC (Water H-class system) by isocratic elution, with mobile phase acetonitrile: ammonium acetate buffer pH 4.5 (20 : 80) and a column Luna® C18 (150 × 4.6 mm i.d.). The flow rate was 0.2 mL/min, and the detection wavelength was 276 nm.

### 2.2. Preparation of Molecularly Imprinted Mesoporous Silica Salbutamol (MIPMS)

The synthesis method uses the direct incorporation method carried out by Jun-Bo et al., with several modifications [20]. MIPMS was synthesized made from several mixtures of salbutamol, CTAB, TEOS, and MTES according to the compositions listed in Table 1.

55.65 mg of salbutamol sulfate and 1.8 g of CTAB were dissolved in 120 mL of water. MTES was added to the mixture solution. After stirring for 30 minutes, TEOS and 3.50 mL of 2M NaOH were added to the mixture. The mixture was stirred for 24 hours. The mixture was filtered, and the solids were washed with water and methanol. Then, it dried in an oven at 80°C for 24 hours. The solid was ground and suspended with acetone. After that, the sorbent was dried in air and washed with 2M HCl in ethanol. Then, it was extracted using the acetic acid solution in methanol (20% v/v) for 24 hours. The solid was dried and washed with methanol and water; washing was repeated until no peak was detected at the maximum wavelength of salbutamol using the UV spectrophotometer. The sorbent was dried at 80°C for 24 hours. The nonimprinted mesoporous silica (NIPMS) was synthesized using the same method but without adding salbutamol.

### 2.3. Physical Characterization

#### 2.3.1. Fourier-Transform Infrared Spectroscopy (FTIR)

Functional groups were determined on the sorbent before and after extraction, using FTIR spectroscopy at wave number 4000–400 cm^−1^. 198 mg of potassium bromide (KBr) and 2 mg of the sorbent sample were ground and printed into plates and then analyzed using FTIR; 200 mg of KBr was used as a blank.

#### 2.3.2. Scanning Electron Microscope (SEM)

Physical characteristics to determine the morphology of the synthesized polymers were carried out using a scanning electron microscope (SEM) [28]. The morphology of the sorbent powder was observed using SEM at a magnification of 10,000x.

### 2.4. Adsorption Capability Evaluation

The adsorption capability evaluation was carried out to analyze the sorbent's swelling ability in different solvents [29]. 5 mL of standard salbutamol 5 ppm solution were added into a vial containing 20 mg of MIPMS or NIPMS. The mixture was shaken and allowed to stand for 24 hrs. After incubation, the filtrate was measured by using a UV spectrophotometer. The amount of salbutamol absorbed was calculated based on the difference between the initial concentration of salbutamol and the final concentration of salbutamol in the filtrate.

### 2.5. Adsorption Capacity Evaluation

Standard salbutamol solution with different concentrations was used to evaluate the adsorption capacity. 5 mL of standard salbutamol solution were added into a vial containing 20 mg of MIPMS or NIPMS. The mixture was shaken and allowed to stand for 24 hrs. After incubation, the filtrate was measured by using a UV spectrophotometer. The amount of salbutamol absorbed was calculated based on the difference between the initial concentration of salbutamol and the final concentration of salbutamol in the filtrate. The results are plotted in the Freundlich and Langmuir adsorption isotherm model.

### 2.6. Application of the SPE Sorbent to Extract Salbutamol from Spiked Serum

#### 2.6.1. Optimization of the SPE Extraction Condition

200 mg of MIPMS-2 and NIPMS-2 were, respectively, placed into the SPE cartridges with frits at either end. Optimization SPE extraction condition was evaluated to determine the conditioning solvent, loading solvent, washing, and elution solvent that can produce the highest %recovery of salbutamol.

#### 2.6.2. Application of the SPE Sorbent

Application of MIPMS and NIPMS was carried out by spiking serum with standard salbutamol solution without other compounds and standard salbutamol solution mixed with standard terbutaline solution. 200 mg of MIPMS or NIPMS was put into a 3 mL SPE cartridge. The cartridge was preconditioned with 1.0 mL methanol, followed by 1.0 mL water. 1.0 mL of the spiked serum was loaded into the MIPMS-SPE or NIPMS-SPE. Then, the cartridge was washed with 3 × 1 mL of acetonitrile: water (1 : 1, v/v). After the washing process, 2 × 1 mL methanol: acetic acid (95 : 5, v/v) was used as the eluent solvent. The 700 *μ*L of the eluent was evaporated using nitrogen and then redissolved with a 700 *μ*L mixture of acetonitrile and water (1 : 1). The sample was analyzed using UPLC.

### 2.7. Nitrogen Adsorption-Desorption Analysis

According to the SPE result, nitrogen adsorption-desorption analyses were performed to see the material's pore size range. Brunauer–Emmett–Teller (BET) theory was used to determine the surface area, and Barrett–Joyner–Halenda (BJH) theory was used to calculate the total pore volume.

## 3. Results and Discussion

### 3.1. Synthesis of MIPMS and NIPMS

In this study, the synthesis was carried out using two ratios of TEOS : MTES as silica and organosilica precursors, i.e., 2 : 1 and 3 : 1. This comparison was selected based on the research of Jun-Bo et al., which stated that the TEOS : MTES ratio of 2 : 1 resulted in a high adsorption capacity, while the TEOS : MTES <2 ratio resulted in a decreased adsorption capacity value [20]. The concentration of TEOS affects the mesoporous structure. A higher concentration of TEOS will produce an irregular mesoporous structure where this mesoporous structure will affect the adsorption ability [21, 30]. In the synthesis of MIPMS, salbutamol sulfate was used as a template molecule, TEOS and MTES as silica and organosilica precursors, and CTAB as a directing agent. In the synthesis process, a surfactant micellar solution was first formed between CTAB and salbutamol sulfate, and then MTES and TEOS were self-hydrolyzed and self-condensed. The two condensed silanes will cocondensate on the micelle surface, and the organic group and Si-OH will bind to the template molecule [20]. This study synthesized NIPMS using the same method without adding a template molecule (salbutamol sulfate) as a control to ensure molecular recognition [31].

**Figure 1:**
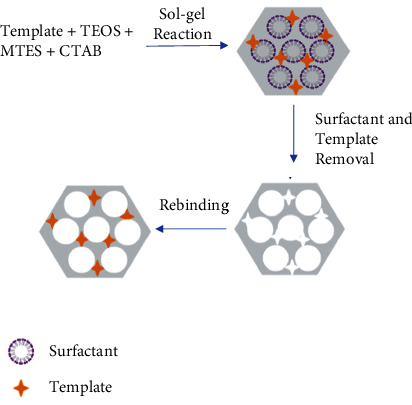
The general synthesis method of molecularly imprinting polymer mesoporous silica (MIPMS).

The Soxhlet extraction method was used to remove CTAB as a surfactant. It was chosen because this extraction process will not remove methyl groups. In contrast, methyl groups will be decomposed on the surface of the sorbent caused to high temperatures in the extraction process if using the calcination process [32]. This methyl group is a functional group provided by the sorbent to interact with the template molecule. The interaction that occurs between the methyl group and the -CH=CH- group in salbutamol is a hydrophobic interaction [33]. This extraction process is not only to remove CTAB but also to remove template molecules. The template molecule was removed to generate the binding site of MIPMS. The mixed solvent of methanol: acetic acid (8 : 2) is used as an extraction solvent. Acetic acid breaks hydrogen bonds between salbutamol and MIPMS, and methanol helps dissolve salbutamol during extraction [34, 35]. The general synthesis method for MIPMS is shown in Figure 1.

### 3.2. Physical Characterization

#### 3.2.1. Fourier-Transform Infrared Spectroscopy (FTIR)

**Figure 2:**
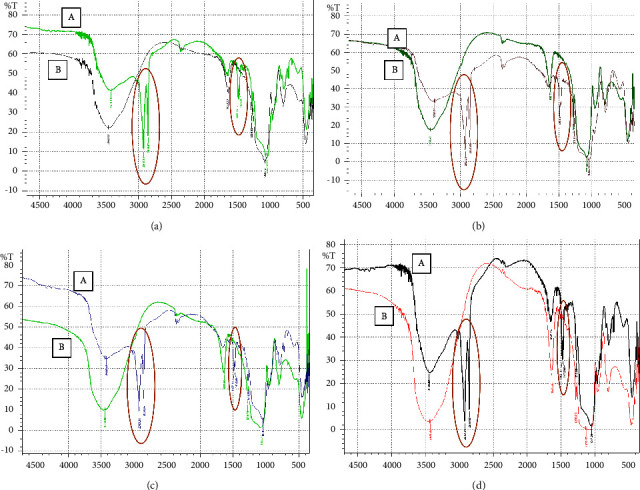
Infrared spectra of MIPMS and NIPMS: (a) A, MIPMS-1 before extraction; B, MIPMS-1 after extraction. (b) A, NIPMS-1 before extraction; B, NIPMS after extraction. (c) A, MIPMS-2 before extraction; B, MIPMS-2 after extraction. (d) A, NIPMS-2 before extraction; B, NIPMS-2 after extraction. Note: the red circle shows the disappearing absorption band after the extraction process.

The synthesized MIPMS and NIPMS were analyzed to identify and characterize the functional groups on the polymer using the FTIR instrument, which was carried out in the wave number range of 400–5000 cm^−1^ [36]. Characterization using FTIR was carried out on MIPMS and NIPMS before and after extraction. The results of the characterization with FTIR can be seen in Figure 2. Based on the results in Figure 2, the functional groups present in CTAB can be observed in MIPMS and NIPMS before extraction, which is indicated by the presence of absorption bands at wave numbers 2919–2921 cm^−1^ and 2849–2851 cm^−1^. The peak in this wave number indicates the presence of symmetrical and asymmetric stretching vibrations from C-H from the methyl and methylene groups. Then, the absorption band at wave number 1472–1488 cm^−1^ indicates the presence of vibrations from the C-N group [18]. After extraction, The FTIR spectra of MIPMS and NIPMS did not show any absorption bands at these wave numbers indicating that CTAB had been extracted.

In the FTIR spectra of MIPMS and NIPMS, before and after extraction, there is absorption at wave numbers 3400–3500 cm^−1^ and 1630–1640 cm^−1^. Absorption at wave numbers 3400–3500 cm^−1^ and 1630–1640 cm^−1^ indicates stretching and bending vibrations of the -OH group on silanol, while wave numbers around 1040–1060 cm^−1^ indicate stretching vibrations from Si-O-Si bonds [37]. The appearance of an absorption band at a wave number of around 1270 cm^−1^ indicates the formation of a Si-C bond which suggests that the methyl group has been bound in the silica structure [33]. The summary of the functional group of MIPMS and NIPMS before and after extraction is shown in Table 2.

#### 3.2.2. Scanning Electron Microscope (SEM)

**Figure 3:**
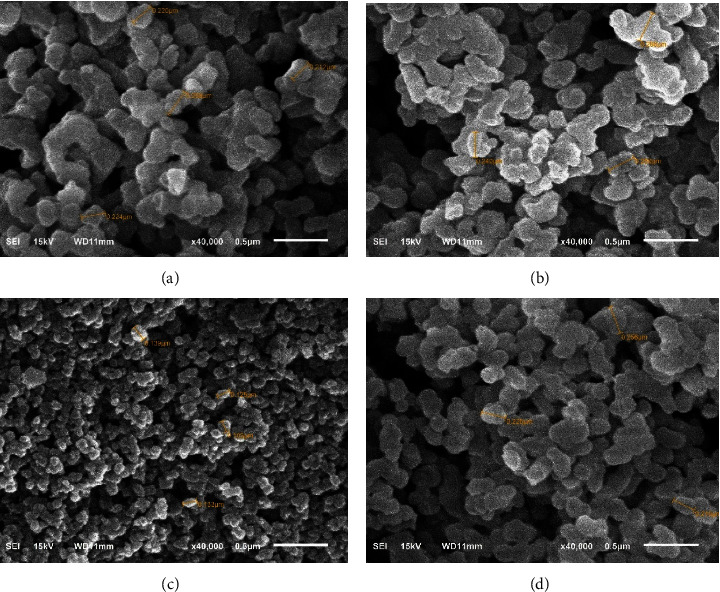
The morphology of MIPMS and NIPMS using SEM: (a) MIPMS-1, (b) NIPMS-1, (c) MIPMS-2, and (d) NIPMS-2.

Scanning Electron Microscopy (SEM) is one of the common methods to determine polymer morphology (Figure 3). All sorbents have the same morphology and are spherical in shape, but MIPMS-2 has a smaller particle size. MIPMS-1, NIPMS-1, and NIPMS-2 have a particle size of about 200–250 nm, while MIPMS-2 has a particle size of around 100–130 nm. All sorbents are included in the nanoparticle size [38]. This difference in particle size can be due to the amount of TEOS used by MIPMS-2 being more than MIPMS-1. The higher concentration of TEOS in the composition of MIPMS-2 makes it have smaller particle size than MIPMS-1 [21]. The imprinting process on MIPMS-2 also causes the resulting particle size to be smaller than that of NIPMS-2. In NIPMS-2, the polymerization process occurs without a pattern due to the absence of a template [39].

### 3.3. Adsorption Capability Evaluation

**Figure 4:**
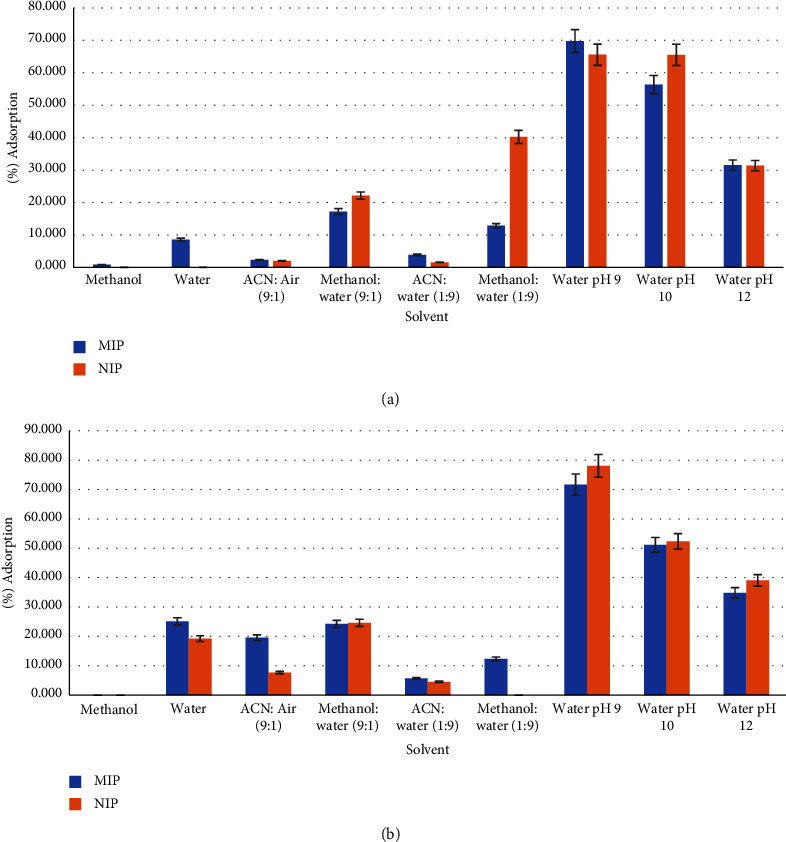
(a) Adsorption capability of MIPMS-1 and NIPMS-1. (b) Adsorption capability of MIPMS-2 and NIPMS-2. Note: ACN = acetonitrile.

In this study, different solvents were used to evaluate the swelling ability of MIPMS. The swelling power of a sorbent can affect the adsorption ability or the recognition of the target compound because when the MIP sorbent swells at a certain level, the size and shape of the imprinted site will change. This geometrical change at the imprinted site may result in a loss of adsorption ability or selectivity of the MIP sorbent [40]. The results of the adsorption capability evaluation of MIPMS-1 and NIPMS-1 can be seen in Figure 4(a). Adsorption capability evaluation of MIPMS-2 and NIPMS-2 can be seen in Figure 4(b).

Based on the adsorption capability data, the highest adsorption capability for MIPMS-1 has reached in water at pH 9 (69.761% ± 1.97%) and NIPMS-1 was 65.525% ± 4.73%, whereas the appropriate solvent for swelling of MIPMS-2 has resulted in water with adsorption capability of about 25.129% ± 0.97% and 19.265% ± 3.63% for NIPMS-2. Water has hydrogen bonds that can prevent the formation of complexes between analytes so that it can increase the interaction of the analyte with the active site [9, 41]. When compared to MIPMS1, MIPMS2 has better adsorption ability in water. This could be due to differences in the TEOS/MTES ratio used. MIPMS1 uses a TOES : MTES ratio of 2 : 1, while MIP-MS-2 uses a TOES : MTES ratio of 3 : 1 so that there are more Si-OH groups which can increase the hydrogen bonds between oxygen atoms of Si-OH in MIP-MS-2 with hydrogen atoms in the -NH and -CH3 groups in salbutamol [42, 43].

### 3.4. Adsorption Capacity Evaluation

Adsorption capacity is one of the essential factors in molecular imprinting. The adsorption capacity was evaluated to see the maximum amounts of targets that could bind with the sorbent [44]. In addition, the purpose of this adsorption capacity was to determine the mechanism of the adsorption interaction between template molecules and MIP [36]. Evaluation of adsorption capacity was determined using the adsorption isotherm model. This method helps describe MIP characteristics and calculate the relationship between bond parameters and affinity distribution [29]. The results of the adsorption capacity evaluation are listed in Table 3.

Based on the results of Table 3, it can be concluded that the Freundlich isotherm model is the appropriate model because the linearity value (*R*^2^) of the Freundlich isotherm model is greater than the Langmuir isotherm [45, 46]. The Freundlich isotherm model shows that adsorption occurs on heterogeneous surfaces with different affinities and has a multilayer adsorption system [47].

In the Freundlich isotherm model, the heterogeneity of the sorbent is calculated exponentially as a log function, which facilitates the calculation by converting it into a linear function [48]. The Freundlich isotherm function uses two test parameters, namely, the values of *a* and *m*. These parameters, respectively, indicate the binding affinity of the polymer and the degree of bond heterogeneity. The degree of homogeneity has a value between 0 and 1. An *m* value equal to one indicates a homogeneous sorbent bind system, i.e., the surface of the polymer particles has the same binding ability, and an *m* value close to zero means a heterogeneous binding system [49]. MIPMS-1 and NIPMS-2 have *m* values close to 1, indicating that the sorbent is more homogeneous than the homogeneity index values of NIPMS-1 and MIPMS-2 (less than 1). The binding affinity value (a) of MIPMS-1 is greater than that of MIPMS-2. However, when compared with NIPMS-1, the affinity of MIPMS-2 is lower. Therefore, based on the significant degree of the binding affinity value between MIPMS and NIPMS, MIPMS-2 and NIP- MS-2 resulted in differences in bind affinity, so this polymer was selected as a sorbent for SPE.

## 4. Application of the SPE Sorbent to Extract Salbutamol from Spiked Serum

### 4.1. Optimization of the SPE Extraction Condition

The 200 mg of MIPMS-2 and NIPMS-2 were packed in cartridges. The optimization of MIP-SPE extraction conditions is determined by selecting the type of solvent for conditioning, loading, washing, and eluting. The SPE cartridges were preconditioned using methanol and then water. Water was used to load 2 ppm salbutamol because the adsorption ability of MIPMS-2 in water gave a good adsorption value. The most critical step in SPE extraction is the washing step. This step maximizes the interaction between the analyte and the binding site, where matrix disturbances can be removed in the SPE without loss of the analyte [23]. Three solvents were used as a washing solvent (Table 4). The methanol: acetic acid (95 : 5) was used as an eluting solvent. The addition of acetic acid in the eluting solvent can interfere with the interaction between the template molecule and the binding site of the sorbent. The acetic acid will replace the template molecule to bind in the binding site [50]. The % recovery of salbutamol using MIPMS-2 as an SPE sorbent is shown in Table 4.

Based on the result, the selected condition is (1) the precondition for using methanol followed by water, (2) loading using salbutamol standard in water, (3) washing solvent using acetonitrile: water (1 : 1), and (4) eluting solvent using methanol: acetic acid (95 : 5).

### 4.2. Application of the SPE Sorbent

The results of % salbutamol recovery with spike salbutamol concentration of 2 ppm were 104.79% ± 1.01% for MIPMS-2-SPE and 87.85% ± 17.75% for NIPMS-2-SPE. The imprinting factor (IF) value obtained is 1.2; the IF value was obtained from the ratio of the amount or percent of sorbate bound to MIPMS with the amount or percent of sorbate bound to NIPMS [51]. A good IF value more than 1 indicates a better MIP imprint site than NIP [52]. Based on the results, the analytical performance of MIPMS-2 is better than that of NIPMS-2.

Zhang et al. have developed an analytical method for analyzing salbutamol in urine and plasma. The SPE using the Sep-Pak Silica column is used as a pretreatment of samples. The recovery of this method reached about 77.32% and 81.25% [53]. Compared with the recovery results in this study, MIPMS-SPE can increase the value of % recovery of salbutamol compared to conventional SPE. Alizadeh and Fard also developed the MIP-base sensor for analyzing salbutamol. They synthesized Cu2+-mediated nanosized salbutamol-imprinted polymer. This method has higher recoveries of about 90–102% for salbutamol in plasma and 94–103% in urine [54]. However, compared to MIPMS-SPE, the pretreatment process in this method requires a long preparation time, is complicated, and requires the right temperature.

The selectivity study was carried out by spiking serum with salbutamol and its analog structure (terbutaline).  Table 5 shows that the % recovery of salbutamol extracted with MIPMS-2 has a higher value than the % recovery from terbutaline, indicating that MIPMS-2 can distinguish the two compounds [20]. However, the IF value for the salbutamol compound is 1.01, while the IF value for the terbutaline compound is 1.21. This result showed that when there are other compounds, the ability of NIPMS-2 increases, which may be due to an increase in nonmolecular interactions in the form of physical interactions [55]. This study has limitations because the selectivity was carried out with only one similar compound, namely, terbutaline. Therefore, selectivity testing with other compounds is needed to conclude that MIPMS is selective or not. Besides that, it is also necessary to optimize the conditions of MIPMS synthesis to produce sorbents with better selectivity so that when salbutamol is present in biological samples with analog compounds, nonmolecular interactions do not occur.

### 4.3. Nitrogen Adsorption-Desorption Analysis

**Figure 5:**
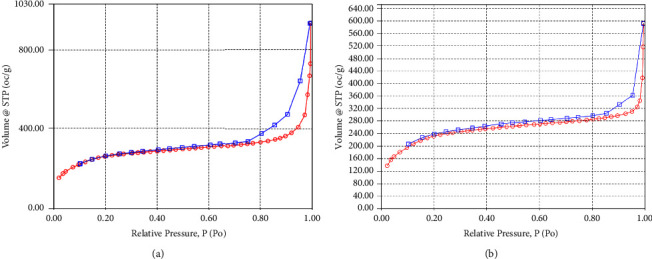
N_2_ adsorption and desorption isotherm curve: (a) MIPMS-2; (b) NIPMS-2.

The curve of the nitrogen gas adsorption-desorption isotherm of MIPMS-2 and NIPMS-2 is shown in Figures 5(a) and 5(b). According to the IUPAC classification, MIPMS-2 and NIPMS-2 exhibit the type IV isotherm, which indicates that the material's pore size is in the mesoporous range [56, 57].

The surface area of the sorbent was measured using the Brunauer–Emmett–Teller (BET) method, and the total pore volume and pore size were measured using the Barret–Joyner–Halenda (BJH) method. The results of the surface area analysis and pore volume can be seen in Table 6. These results indicate that MIPMS-2 has a higher surface area (870.492 m^2^/g) than NIPMS-2 (779.026 m^2^/g). In addition, the total pore volume of MIPMS-2 has a higher value (1.066 cm^3^/g) than that of NIPMS-2 (0.561 cm^3^/g). The larger surface area shows the effect of the printing process forming imprinted pores or binding cavities [47, 58]. With increasing surface area and pore volume, the adsorption ability of a sorbent also increases [59].

## 5. Conclusion

The sorbent of MIPMS has been synthesized using the direct incorporation method. MIPMS-2 has better recognition and binding affinity for salbutamol than NIPMS-2. The adsorption capacity of MIPMS-2 was 0.0934 mg/g, and the % recovery result of salbutamol in the serum sample was about 104.79% ± 1.01%. However, further studies are needed to optimize MIPMS synthesis conditions in order to increase the imprinting factor and selectivity of MIPMS.

## Figures and Tables

**Table 1:** Composition of materials used in the synthesis of MIPMS and NIPMS.

Sorbent	Composition			
	Salbutamol sulphate	CTAB (g)	TEOS (mL)	MTES (mL)
MIPMS-1	55.6 mg	1.8	2.511	1.109
NIPMS-1	—	1.8	2.511	1.109
MIPMS-2	55.6 mg	1.8	3.763	1.109
NIPMS-2	—	1.8	3.763	1.109

**Table 2:** MIPMS-2 and NIPMS-2 characterization results using FTIR.

Wave number (cm^−1^)								Functional groups
MIPMS-1		NIPMS-1		MIPMS-2		NIPMS-2		
Before extraction	After extraction	Before extraction	After extraction	Before extraction	After extraction	Before extraction	After extraction	
3413.10	3458.43	3392.85	3458.43	3428.53	3445.89	3446.85	3434.32	-OH stretching
2919.31	—	2922.21	—	2919.31	—	2919.31	—	C-H stretching symmetries
2849.87	—	2851.80	—	2850.84	—	2849.87	—	C-H stretching asymmetries
1653.99	1653.99	1632.77	1632.77	1635.66	1653.66	1647.24	1635.66	-OH bending
1487.14	—	1490.04	—	1487.14	—	1487.14	—	C-N
1271	1278.83	1271.11	1278.83	1272.06	1279.79	1272.08	1278.83	Si-C
1045.44	1080.16	1054.44	1079.63	1045.44	1074	1047.36	1122.59	Si-O

**Table 3:** Results of adsorption capacity evaluation using Freundlich isotherm and Langmuir isotherms.

Sorbent	Freundlich isotherm			Langmuir isotherm		
	*a* (mg/g)	*M*	*R * ^2^	qm (mg/g)	Ke	*R * ^2^
MIPMS-1	0.6318	1.1560	0.9147	−4.2790	−0.1258	0.3073
NIPMS-1	1.0940	0.6486	0.8352	−14.7710	−0.0586	0.1361
MIPMS-2	0.0934	0.5345	0.997	0.5873	0.1203	0.9396
NIPMS-2	0.0407	1.1316	0.9953	−2.6015	−0.0177	0.6295

Note: *a* = adsorption capacity (mg/g); *m* = homogeneity index; qm = adsorption capacity (mg/g); Ke = Langmuir's constant; *R*^2^ = correlation coefficient.

**Table 4:** % recovery salbutamol using different washing solvents.

Washing solvent	% recovery
Acetonitrile	107.59% ± 13.63%
Acetonitrile : water (1 :1)	100.843% ± 2.21
Acetonitrile : water (3 : 7)	105.344 ± 4.51

**Table 5:** % recovery of salbutamol and terbutaline in serum spike samples.

Sorbent	% recovery	
	Salbutamol (%)	Terbutaline
MIPMS-2	108.91 ± 3.16	89.79 ± 3.78
NIPMS-2	107.64 ± 6.83	73.93 ± 1.44

**Table 6:** Surface area and total pore volume of MIPMS-2 and NIPMS-2.

Sorbent	Surface area BET (m^2^/g)	Total pore volume BJH (cm^3^/g)
MIPMS-2	870.492	1.066
NIPMS-2	779.026	0.561

## Data Availability

The data used to support the findings of this study are available from the corresponding author upon request.
